# Functional and cellular characterization of human Retinoic Acid Induced 1 (RAI1) mutations associated with Smith-Magenis Syndrome

**DOI:** 10.1186/1471-2199-11-63

**Published:** 2010-08-25

**Authors:** Paulina Carmona-Mora, Carolina A Encina, Cesar P Canales, Lei Cao, Jessica Molina, Pamela Kairath, Juan I Young, Katherina Walz

**Affiliations:** 1 John P. Hussman Institute for Human Genomics, Miller School of Medicine, University of Miami, Miami, Florida, USA; 2 Centro de Estudios Científicos, CECS, Valdivia, Chile; 3 Universidad Austral de Chile, Valdivia, Chile; 4 Centro de Ingeniería de la Innovación (CIN), CECS, Valdivia, Chile

## Abstract

**Background:**

Smith-Magenis Syndrome is a contiguous gene syndrome in which the dosage sensitive gene has been identified: the Retinoic Acid Induced 1 (*RAI1*). Little is known about the function of human RAI1.

**Results:**

We generated the full-length cDNA of the wild type protein and five mutated forms: *RAI1-HA *2687delC, *RAI1-HA *3103delC, *RAI1 *R960X, *RAI1-HA *Q1562R, and *RAI1-HA *S1808N. Four of them have been previously associated with SMS clinical phenotype. Molecular weight, subcellular localization and transcription factor activity of the wild type and mutant forms were studied by western blot, immunofluorescence and luciferase assays respectively. The wild type protein and the two missense mutations presented a higher molecular weight than expected, localized to the nucleus and activated transcription of a reporter gene. The frameshift mutations generated a truncated polypeptide with transcription factor activity but abnormal subcellular localization, and the same was true for the 1-960aa N-terminal half of RAI1. Two different C-terminal halves of the RAI1 protein (1038aa-end and 1229aa-end) were able to localize into the nucleus but had no transactivation activity.

**Conclusion:**

Our results indicate that transcription factor activity and subcellular localization signals reside in two separate domains of the protein and both are essential for the correct functionality of RAI1. The pathogenic outcome of some of the mutated forms can be explained by the dissociation of these two domains.

## Background

A large fraction of genome variation between individuals is comprised of submicroscopic copy number variation of DNA segments (CNVs) [1-6]. Genomic disorders are the clinical manifestation of pathological CNV. They are frequent conditions (~1 per 1,000 births) and often sporadic resulting from *de novo *rearrangements [7]. In a subset of such conditions the rearrangements comprise multiple unrelated contiguous genes that are physically linked and thus have been referred to as contiguous gene syndromes (CGS). An increasing number of CGS are being described, each of them presenting a complex and specific phenotype. Although several genes are usually present in the segmental aneuploidy; only a small subset of them conveys phenotypes as a function of copy number alteration. These particular genes are referred to as "dosage sensitive genes".

The Smith-Magenis Syndrome, SMS, (OMIM# 182290) is a CGS associated with a microdeletion within chromosome 17 band p11.2. SMS was first described in 1986 with a birth prevalence estimated at 1/25,000. The clinical phenotype includes craniofacial abnormalities, brachydactyly, self injurious behavior, sleep abnormalities and mental retardation. Less commonly reported is cleft palate, congenital heart defects, seizures, hearing impairment and urinary tract anomalies [8]. Molecular studies revealed a common deleted region of ~4 Mb in the majority of SMS patients (>70-80%) [9-11]. Unusual sized deletions (smaller or larger) were observed in 20-25% of patients [12-14]. By examining the breakpoints in unusual sized deletions, the SMS critical region was redefined to a ~950 kb interval, in which 15 genes and eight predicted genes were present [11-14]. Retinoic Acid Induced 1 gene (*RAI1*) is located in the middle of the SMS critical region. Point mutations (nonsense and frameshift as well as missense alleles) in *RAI1 *were identified in patients with clinical presentation of SMS but no molecular deletion found by FISH [15-18], suggesting that *RAI1 *is the dosage sensitive gene causative of SMS. Studies on mouse models [19] and humans [20,21] indicate that *RAI1 *is likely the dosage sensitive gene responsible for clinical features in the Potocki-Lupski Syndrome (PTLS), (OMIM# 610883) a neurobehavioral disorder with autistic features that is caused by reciprocal duplication of the 17p11.2 region [20-23]. The *RAI1 *gene consists of six exons [24] that span over 120 kb. The third exon contains >90% of the coding region and it is within this exon where all mutations have been identified to date.

Little is known about the cellular and developmental role of RAI1. Expression of GT1, a splice variant of *Rai1 *was markedly up-regulated by treatment with retinoic acid in a mouse carcinoma cell line P19 [25]. A polymorphic CAG repeat is present in the N-terminus of the RAI1 protein, the length of which is associated with the age of onset of spinocerebellar ataxia type 2 [26] and the response to neuroleptic medication in schizophrenia [27]. Moreover, RAI1 was recently associated with non syndromic autism [28]. Bioinformatic analyses have suggested that RAI1 might be a transcriptional regulator [15,16]. RAI1 contains several patches of 50% similarity with TCF20, a transcriptional cofactor, and these two genes have a similar gene structure. RAI1, in both human and mouse, have two putative bipartite nuclear localization signals (NLSs) predicted *in silico*, and possesses a zinc finger like plant homeo domain (PHD) in the C-terminus, which is also present in the trithorax family of chromatin remodeling transcriptional regulators [16]. *In vitro *studies of the murine Rai1 protein indicated that it can be transported to the nucleus and has transactivation activity [29]. However, no systematic analysis of the human RAI1 protein has been done. Here we describe the characterization of the wild type RAI1 protein, plus five mutated forms of the protein, including four that have been associated with the SMS clinical phenotype. Subcellular localization of the resulting proteins and transcription factor activity were studied for the wild type and mutant forms. Our results indicate that the transcription factor activity and subcellular localization of the protein are essential for the pathogenic outcome of some of the mutated forms.

## Results

### The transcription factor activity of the murine Rai1 protein is dependent on the cell type

**Figure 1:**
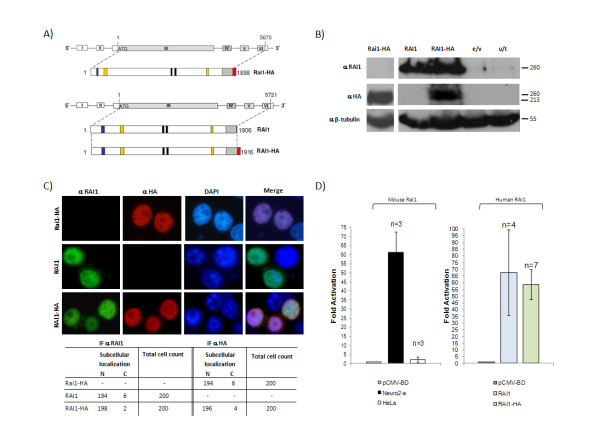
**Generation of murine and human -HA tagged RAI1 and molecular evaluation of the resulting proteins**. **A) **Schematic representation of mouse (Rai1) and human (RAI1) genomic and protein structure. In blue is represented the Poly-Gln domain, in yellow: Poly-Ser domains, in black: *in silico *described NLS, in slanted lines: the PHD domain. The coding sequence for HA epitope (represented in red) was added by PCR at the 3' end of full-length cDNA. **B) **Cells transfected with either mouse *Rai1-HA *plasmid (Rai1-HA), the human *RAI1 *plasmid, *RAI1 *or *RAI1-HA*, an empty vector (e/v) or untransfected cells (u/t) were lysed and a western blot analysis was performed with an anti HA antibody (αHA). The molecular weight of the resulting proteins is depicted. The anti β-tubulin antibody (α β-tubulin) was used as loading control. **C) **Neuro-2a cells were transiently transfected with the mouse *Rai1-HA *plasmid (Rai1-HA), the human *RAI1 *and *RAI1-HA *plasmids. Immunofluorescence with an antibody that recognized the HA tag (αHA) (red) and an antibody that recognizes the first 30 aa of RAI1 (αRAI1) (green) are shown. Untransfected cells in the same slide were used as negative control. Nuclei were stained with DAPI. The table represents the subcellular localization observed for 200 counted cells positive for immunodetection. **D) **Transactivational activity. The fold of luciferase activation is represented for the empty vector (gray), Neuro-2a cells transfected with mouse *Rai1-HA *(black), human *RAI1 *(light blue), and *RAI1-HA *(light green) and HeLa cells transfected with *Rai1-HA *(white). Values represent mean +/- SEM.

Human and mouse RAI1 share more than 80% in DNA and amino acid homology sequence. Moreover, several mouse models for SMS were developed and studied, and they presented similar phenotypes to those found in the SMS patients, reinforcing the idea of a comparable function for murine and human RAI1 [30]. A previous report by Bi *et al*., [29] showed that the murine Rai1 protein presented a mild transcription factor activity and a nuclear subcellular localization. In order to corroborate previous findings and to set up the optimal conditions for our studies of human RAI1, we subcloned the murine *Rai1 *full-length cDNA kindly donated by Dr. James Lupski, into different vectors that allowed us to see the molecular weight, subcelullar localization and transcription factor activity of the murine protein (figure 1). The expected 201.5 kDa molecular weight for the murine protein was similar to the obtained molecular weight (213 kDa) (figure 1B), as well as the nuclear subcellular localization (figure 1C). It was previously reported that Rai1 has relatively weak transactivation activity in transfected HeLa cells in comparison to other transcription factors [29]. Neurobehavioral abnormalities are the prevalent clinical presentation in SMS patients. Based on this we wanted to examine whether Rai1 has a different transactivation activity in cells derived from murine neuroblastoma, the Neuro-2a cell line. In order to do this, full-length *Rai1 *was fused with the GAL4 DNA binding domain (GAL4-BD) and co-transfected into HeLa and Neuro-2a cells with a luciferase reporter plasmid which contains five tandem repeats of yeast GAL4-binding sites upstream of the luciferase gene, plus a plasmid containing the beta galactosidase gene to control transfection efficiency. The results showed that the transcription factor activity in HeLa cells is 2.2 +/- 1.5 folds over the empty vector and in Neuro-2a cells is 61.3 +/- 11.2 indicating that the murine protein has a transactivational activity several times stronger in the Neuro-2a cell line than in HeLa cells, suggesting that there is a specific machinery in neuronal cells that may be related to Rai1 transcription factor activity (figure 1D).

### Human RAI1 is a nuclear protein with transcription factor activity

In a first attempt to understand and characterize the function of human RAI1 we generated the full-length cDNA coding for the wild type protein. The full-length cDNA was obtained as described in Material and Methods. The final full-length sequence is the same as the one reported in NM_030665 with SNP A/G at position 1992. In order to have an easy way to identify the transfected RAI1 from the endogenous protein, an HA tag was added in the 3' end (figure 1A). Since we found that Rai1 activity was dependent of cellular type, we performed all our studies in mouse Neuro-2a neuroblastoma cells. Neuro-2a cell line was preferred over the SH-SY5Y (human neuroblastoma) cell line despite its murine origin, since the human ones grow at an extremely slow rate and making them very difficult to transfect. In order to determine the molecular weight of the protein, both clones the *RAI1 *and *RAI1-HA *cDNA were transiently transfected, and 48 h post-transfection the cell lysate was run in a 10% SDS-PAGE gel. The predicted *in silico *molecular weight for RAI1 is 203 kDa. As can be observed in figure 1B, the molecular weight of both proteins is ~260 kDa, and the addition of the HA tag is not inducing instability of the resulting protein. The difference between the molecular weight predicted and the obtained could be explained by post-translational modifications (PTM) for example glycosylations. We found several predicted glycosylation sites on the first amino-terminal half of RAI1 (analysis in NetOGlyc 3.1 server, EXPASY Tools, http://www.expasy.org) [31].

To prove if the human protein has transcription factor activity, we subcloned the *RAI1 *and *RAI1-HA *full-length cDNA into a GAL4-binding domain vector (pCMV-BD, Stratagene). It is important to note that the presence of the GAL4-binding domain is enough to localize the protein to the nucleus [32]. Transfection of each clone plus the reporter gene was carried out and 48 h post-transfection the cells were lysed and the activity of the luciferase reporter gene was measured. As can be seen in figure 1D, the expression of the wild type RAI1 and RAI1-HA derives in an 67.5 +/- 31.8 and 58.8 +/- 11.1 fold increase of the luciferase activity respectively, indicating that the human protein presented transcription factor activity and that the addition of the HA tag did not interfere with the activity of the resulting protein. RAI1 has a bipartite nuclear localization signal predicted by informatics analysis [15]. Nuclear subcellular localization was found for both, the RAI1 and RAI-HA proteins (figure 1C) at 36 h post-transfection and this result was confirmed with two different antibodies, one that recognized an epitope within the first 30 amino acids in the N-terminus portion of the RAI1 protein and the anti HA antibody, which recognized the C-terminus end in the RAI1-HA clone. These results indicate that the human RAI1 protein, with or without the HA tag, has a nuclear localization and the ability to activate the transcription of a reporter gene.

### The N-terminal truncated RAI1 polypeptides retain the transcription factor activity but localize in the cytoplasm

**Figure 2:**
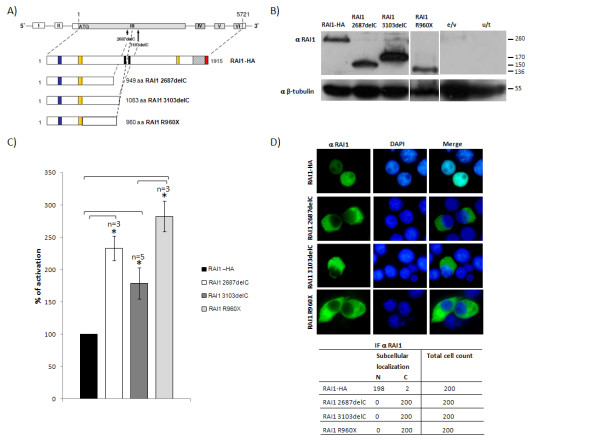
**Evaluation of truncated proteins**. **A) **Schematic representation of the two truncated RAI1 proteins generated by the deletion of a C in positions 2687 and 3103, plus RAI1 R960X. In blue is represented the Poly-Gln domain, in yellow: Poly-Ser domains, in black: *in silico *described NLS, in slanted lines: the PHD domain. The coding sequence for HA epitope is represented in red. **B) **Molecular weight for all truncated proteins was calculated by western blot analysis utilizing anti RAI1 antibody in transfected Neuro-2a cells. The molecular weight obtained for 2687delC, 3103delC and R960X is indicated. e/v: cells transfected with empty vector; u/t: untransfected Neuro-2a cells. **C) **The percentages of activation for the proteins 2687delC (white), 3103delC (grey) and R960X (light grey) are represented. The wild type (black) is considered as 100% of transcription activity. Values represent mean +/- SEM. (*: *P *≤ 0.01). **D) **Immunofluorescence was performed with anti RAI1 antibody (green). Nuclei were stained with DAPI. The table represents a summary of the subcellular localization found in 200 cells. α = antibody against RAI1.

Most Smith-Magenis cases present a deletion in chromosome 17p11.2 that causes the haploinsufficiency of the *RAI1 *gene. However, in some patients heterozygous mutations have been found within the *RAI1 *coding region. In order to study how these mutations can be detrimental we analyzed two independent deletions of one C within the coding region, the *RAI1 *2687delC (not found yet in any patient and spontaneously originated in our laboratory) and the *RAI1 *3103delC [18] (figure 2A). Theoretically in both cases the deletion of a C within the C tract will result in a frameshift, misincorporation of amino acids and a subsequent premature stop codon. In the case of RAI1-HA 2687delC a misincorporation of 53 amino acids followed by a stop codon is expected, while in the RAI1-HA 3103delC protein the misincorporation will be 28 amino acids. In order to avoid the possible interference of the misincorporated amino acids in the following studies we also generated the RAI1 R960X mutation [18], where an arginine is replaced by a stop codon in the coding sequence generating a truncated protein, with no addition of extra amino acids. In order to determine the molecular weight of the resulting proteins, the three clones were transiently transfected, and 48 h post-transfection the cell lysate was run in a 10% SDS-PAGE gel. The predicted molecular weights for the proteins originated by *RAI1-HA *2687delC, *RAI1-HA *3103delC and *RAI1 *R960X are 102, 114 and 103 kDa respectively. As can be observed in figure 2B, the obtained molecular weight of the three truncated proteins was ~150, 170 and 136 kDa respectively. These differences coincide with the predicted versus observed molecular weight differences found for the wild type protein with or without the HA tag, suggesting that the alleged modifications reside in the N-terminal half of RAI1.

We evaluated the transcription factor activity for the truncated products by subcloning the three mutant forms cDNA into the pCMV-BD vector and posterior transfection of each clone plus the reporter gene into Neuro-2a cells. Forty eight hours post-transfection the cells were lysed and the activity of the luciferase reporter gene was measured. As can be seen in figure 2C, all truncated proteins gave an increment of 233.2 +/- 19.2 (for RAI1 2687delC), 179.1 +/- 24.1 (for RAI1 3103delC), and 282.5 +/- 23.8 (for RAI1 R960X), in percentage of activation in comparison with the 100% of the wild type, indicating that the truncated polypeptides retained the transcription factor activity. All the truncated proteins showed a significant increased percentage of transcription activation when compared to the wild type protein (P value ≤ 0.01). The following step was to evaluate the subcellular localization of the truncated fragments in the absence of the GAL4-binding domain since the presence of this domain is enough to localize any protein to the nucleus [32]. The resulting truncated polypeptides would not include the hypothetical Nuclear Localization Signals (NLSs). Cytoplasmatic subcellular localization was found for all the

RAI1-HA 2687delC, RAI1-HA 3103delC and RAI1 R960X polypeptides (figure 2D). All together these results indicate that RAI1-HA 2687delC, RAI1-HA 3103delC and RAI1 R960X are generating truncated polypeptides that retain the transcription factor activity. However, they are not localized in the nucleus but in the cytoplasm of cells.

### Nuclear localization signals are situated in the C-terminus of the human RAI1 protein

**Figure 3:**
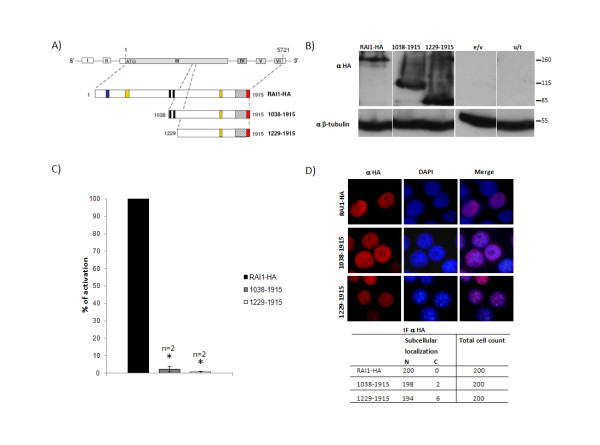
**Evaluation of the proteins generated containing the C-terminal half of RAI1**. **A) **Schematic representation of the construction of proteins 1038-end and 1229-end that were generated by PCR. Both proteins are tagged with the HA peptide on the C-terminal end. In blue is represented the Poly-Gln domain, in yellow: Poly-Ser domains, in black: *in silico *described nuclear localization signals, in slanted line: the PHD domain. **B) **The molecular weight for both proteins was obtained by transfecting them in Neuro-2a cells and then a western blot analysis was performed with anti HA antibody. Molecular weight for the proteins 1038-end and 1229-end are depicted and also are shown the controls with only the transfection of empty vector (e/v) and untransfected Neuro-2a cells (u/t). **C) **The graphic represents the percentage of activation for 1038-end protein (grey) and 1229-end protein (white) compared to the wild type full-length protein (black). Values represent mean +/- SEM. (* depicts statistically significant differences, p ≤ 0.0002). **D) **Each plasmid was transfected in Neuro-2a cells and an immunofluorescence was performed with anti HA antibody. Nuclei staining were made with DAPI. The table shows subcellular localization for 200 cells positive for anti HA. α = antibody against HA.

In order to verify that the C-terminal portion of the RAI1 protein is the one retaining the nuclear localization signals we generated two different fragments, one beginning at Met 1038 and the other beginning at Met 1229 of the RAI1 protein. The difference between them contains the sequence that was assigned as the bipartite NLS by *in silico *methods [15]. Both C-terminal halves were generated as described in Material and Methods (figure 3A). In order to determine the molecular weight of the resulting proteins, both clones were transiently transfected, and 48 h post-transfection the cell lysate was run in a 10% SDS-PAGE gel. As can be observed in figure 3B, the molecular weight of both truncated proteins was ~115 kDa and 85 kDa respectively as predicted; and interestingly these results differ from those obtained with the other proteins analyzed further supporting the possibility of a specific PTM in the N-terminal half of RAI1. We evaluated the transcription factor activity for the C-terminal halves of RAI1 by subcloning them into the pCMV-BD vector with posterior transfection of each clone plus the reporter gene into Neuro-2a cells. As can be seen in figure 3C, both C-terminal halves of RAI1 were unable to activate the transcription of the luciferase reporter gene, since no luciferase activity could be measured in any of the assays. To evaluate the subcellular localization of the truncated fragments, both halves were transfected into Neuro-2a cells and the subcellular localization assayed with anti HA antibody. As can be observed, both halves were found in the nucleus suggesting the presence of extra NLS (figure 3D). Altogether these results indicate that the N-terminal region of the human protein has the transcription factor activity and it is in the C-terminal half that the nuclear localization signaling resides.

### No alterations were found for RAI1 Q1562R and RAI1 S1808N mutants

**Figure 4:**
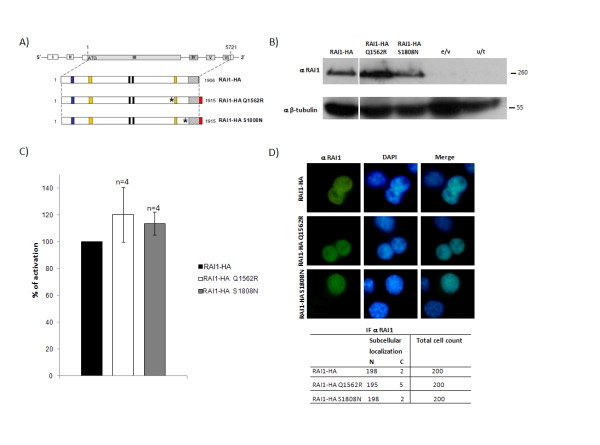
**Molecular evaluation of two point mutations associated with SMS**. **A) **Schematic representation RAI1 Q1562R and RAI1 S1808N. In blue is represented the Poly-Gln domain, in yellow: Poly-Ser domains, in black: *in silico *described nuclear localization signals, in slanted line: the PHD domain. The coding sequence for HA epitope is represented in red. **B) **The molecular weight of mutated proteins was calculated in Neuro-2a cells by western blotting with anti RAI1 antibody. The obtained molecular weight is depicted and also the controls for the immunoreactivity are shown (e/v: extracts transfected only with the empty vector and u/t represents untransfected cells control). **C) **The percentage of the reporter transcription activation is shown for RAI1-HA Q1562R (white) and RAI1-HA S1808N (grey) compared to RAI1-HA wild type protein (black). Values represent mean +/- SEM. **D) **Each plasmid was transfected in Neuro-2a cells and immunofluorescence was performed with anti RAI1 antibody and nuclei staining is shown with DAPI. The table represents subcellular localization of 200 cells immunodetected with anti RAI1 antibody. α = antibody against.

As we mentioned earlier, there are only rare cases with patients with Smith-Magenis Syndrome associated with heterozygous missense mutations in the *RAI1 *coding region. In two of these cases, a missense mutation was found: *RAI1 *Q1562R and *RAI1 *S1808N [17]. In order to investigate if these mutations were affecting the stability, subcellular localization or transcription activity function of the RAI1 protein we generated both mutations as described in Material and Methods (figure 4A). To determine the molecular weight of the resulting proteins, both clones were transiently transfected, and 48 h post-transfection the cell lysate was run in a 10% SDS-PAGE gel. As can be observed in figure 4B, the molecular weight of both proteins is ~260 kDa similar to the wild type protein. Moreover, none of the mutations seem to give the protein any instability, since the relative signal for both mutants and the wild type RAI1 protein utilizing β-tubulin as loading control protein is the same for all of them. Although there could be differences between transfection efficiency of the samples, we have found by densitometric analysis of bands from different western blots (n = 3), that the ratio RAI1/β-tubulin is the same for wild type, RAI1-HA Q1562R and RAI1-HA S1808N (~1 in all cases) (data not shown).

For both mutants we evaluated the transcription factor activity, in the same way that was previously done for the other proteins. As can be seen in figure 4C, both RAI1-HA Q1562R and RAI1-HA S1808N mutated proteins can give an increment of 120.5 +/- 20.4 and 113.7 +/- 8.6 percentage of activation of luciferase activity respectively, when compared with the wild type as 100%. This is similar to what was found for the wild type RAI1-HA protein, indicating that the mutated polypeptides retained the transcription factor activity. We next evaluated the subcellular localization of the mutated proteins, and nuclear localization was found for both of them (figure 4D). All together these results indicate that RAI1 Q1562R and RAI1 S1808N mutations are not affecting the stability, transcription factor activity or subcellular localization of the protein.

## Discussion

Alterations in the *RAI1 *gene have been associated with Smith-Magenis and Potocki-Lupski Syndromes whose clinical presentation includes autistic features, obsessive-compulsive behaviors, attention deficit, developmental delay, mental retardation, EEG abnormalities, sleep disturbances, self-injurious and maladaptive behaviors, among others [19-21]. In addition to this, *RAI1 *gene was associated with spinocerebellar ataxia (SCA2) [26], neuroleptic response in patients with schizophrenia [27], neurobehavioral traits manifested in SMS patients with *RAI1 *loss-of-function alleles [33-46] and non syndromic autism [28]. Despite its relationship with these important traits, there is lack of information about the molecular function of human RAI1.

Several heterozygous nucleotide variations in *RAI1 *gene have been found in SMS patients without 17p11.2 deletions. These mutations include nonsense and missense mutations besides deletions of one or multiple nucleotides [15-18,43].

Within the *RAI1 *gene coding sequence there are several mononucleotide repeats. Intriguingly all five single base pair frameshift mutations found in the *RAI1 *gene associated with the SMS phenotype were in the C-tracts although the number of G-tracts in the *RAI1 *coding region is comparable with the C-tracts [18]. Interestingly, in our laboratory a spontaneous deletion of a C in a four C-tract at the position 2687 occurred, providing further evidence that polyC-tract may be a preferential target for frameshift mutations in *RAI1*.

Here we generated and studied the full-length cDNA of human wild type *RAI1 *and five mutated forms of the protein. Four of them previously associated with SMS. As deletions appear to be a very frequent event in the *RAI1 *coding region, we also included in this study the spontaneously generated 2687delC. In order to complement these studies, we have constructed two smaller proteins corresponding to the residues 1038-1906 and 1229-1906.

It was previously reported that mouse Rai1 has moderate transactivational activity in HeLa cells [29]. Here we confirmed those previous findings and showed that Rai1 has a stronger transactivation activity in Neuro-2a cells, the neuronal derived cell line. These results suggest specificity in the transcription machinery that RAI1 could be part of and further indicate the importance of its role in neuronal derived tissues, since *RAI1 *is a dosage sensitive gene involved in neurobehavioral phenotypes.

Here we report that the wild type human RAI1 protein has transcription factor activity. Our studies of truncated proteins associated with 2687delC and 3103delC frameshifts plus R960X mutant, showed transcription factor activity significantly higher than the wild type protein for the resulting polypeptides. Moreover, the analysis of the two smaller proteins corresponding to the residues 1038-1906 and 1229-1906 showed no capability of activating transcription themselves. According to our results, the human RAI1 showed that the most important region of the protein for stimulating transcription is the N-terminal half, until residue 1034. From the residue 1038 to the end of RAI1 a fragment able to regulate the transactivational activity of the protein or the machinery associated may be present, since all truncated proteins showed a significant increase in transcription activity when compared to the full-length protein. In the N-terminal region, corresponding approximately to the first half of the protein, there is a homology of ~84% between human and murine RAI1. For the murine Rai1 protein, putative transactivation domains were mapped by fusion of different regions to GAL4-BD [29]. The polypeptides containing residues 1-582 or encompassing residues 583 to 1142 have been shown to have transactivation activity while no transactivation activity was found in the region containing the remaining half; consistent with what is reported here for the human protein.

By bioinformatics analysis two putative bipartite nuclear localization signals (NLSs) were defined for RAI1 at positions 1113 and 1176 aa respectively [15]. Despite that we cannot discard the participation of the previously described NLS in the nuclear localization of the RAI1 protein, our results indicate that there is a nuclear localization signal that resides between 1229 aa to the end since the two smaller proteins 1038-1906 and 1229-1906 were able to localize in the nucleus; while the two N-terminal polypeptides were retained in the cytoplasm. These results suggest the presence of another fragment within the C-terminal half of RAI1 that modulates the nuclear localization of the protein. Accordingly, *in vitro *studies of the murine Rai1 protein indicated that it can be transported to the nucleus and that the nuclear localization signals reside between the 1134-1164 and 1203-1229 aa. Besides, on the C-terminal half of Rai1 there are other regions that are also able to transport the protein to the nucleus [29].

**Figure 5:**
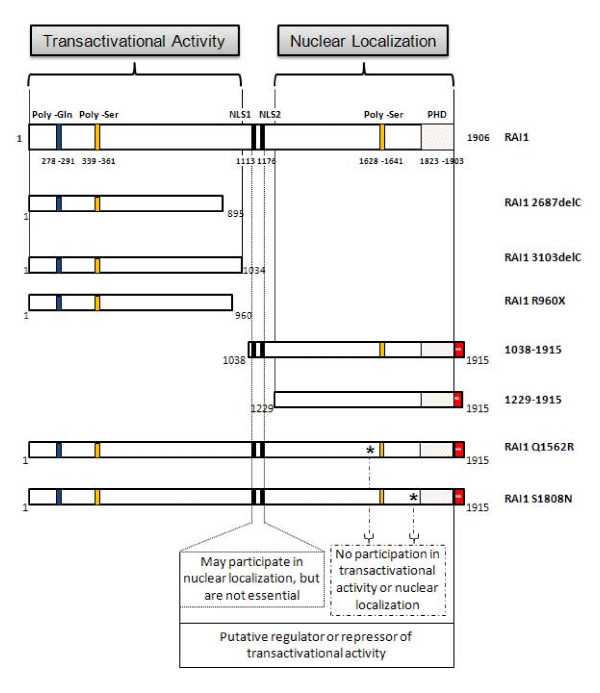
**Summary of the results and definition of two domains in the structure of RAI1**. By *in silico *analyses, several domains have been found for RAI1: a polyglutamine tract at the N-terminal of the protein (in blue), two polyserine domains (in yellow), a PHD domain at the C-terminal of RAI1 (in slanted lines) and two putative nuclear localization signals (in black). The schematic representation of all the mutants analyzed in this study is shown. An asterisk represents the missense mutations. Two defined domains are depicted.

Taking these results together, we can define two clear domains in RAI1: an N-terminal half critical for its transactivational activity and a C-terminal half responsible for its nuclear transportation. In addition, to the C-terminus of both human and mouse RAI1, a zinc finger like plant homeo domain (PHD), which is also present in the trithorax family of chromatin remodeling transcriptional regulators was defined [16]. All these different domains are depicted in figure 5.

The truncated proteins generated by mutations 2687delC, 3103delC and R960X showed to be functional and moreover, they have an increased capability for activating the transcription when compared to the wild type protein. However, none of the resulting polypeptides were able to localize in the nucleus, suggesting that this inability (even retaining transcription factor activity) would be the main pathological way of action for these mutations.

Finally, there are spare cases of SMS patients with mutations in the *RAI1 *gene. We generated two independent mutations, RAI1 Q1562R and RAI1 S1808N. No abnormal molecular weight, subcellular localization or transcription factor activity were found for any of these mutants, suggesting that they may be implicated in the association with other proteins or some other functional role of RAI1 that is not known to date.

## Conclusion

We were able to demonstrate that the human wild type protein has transcription factor activity, and is able to localize to the nucleus. Moreover, we could define a transcription factor domain within the first 1034 aa and that the nuclear localization signals reside in the second half the protein. Altogether the analysis of the mutant proteins indicates that both halves are necessary for the correct subcellular localization and function of the protein, and that other molecular/functional role of RAI1 remains unknown.

## Methods

### Plasmid Constructs

The full-length clone of murine *Rai1 *was kindly provided by Dr. James Lupski, Department of Molecular & Human Genetics, Baylor College of Medicine, Houston, Texas, USA. The sequence of influenza virus hemagglutinin epitope (HA) was added to the 3' extreme to generate a tag by PCR using pCR3.1 *Rai1 *as template, with the primers *Forward*: GCCTCATCCTGAGAAGCAAC and *Reverse*: AAGTCTAGATTAAGCGTAATCTGGAACATCGTATGGGTACAACGGCAGCCTCTTATGTTTG. The reverse primer contains the last 22 nucleotides of *Rai1 *cDNA without its stop codon, the coding sequence of hemagglutinin epitope, followed by a stop codon and an *Xba*I restriction site. The product of the reaction was purified and ligated into pGEM-T easy vector (Promega) and sequenced. In order to obtain the full-length *Rai1-HA *clone this PCR fragment was subcloned into the pCR3.1 *Rai1 *between the *Acc*I and *Xba*I sites.

As a first step to obtain the complete *RAI1 *cDNA a PCR product was generated by amplification of a human brain cDNA library with the following primers: *Forward*: GGCCTGGTAAATGTGGGCACCGGG and *Reverse*: AAGGCGGCCGCTTAAGCGTAATCTGGAACATCGTATGGGTACGGCAGCCTCTTATGTTTGGGAC. The reverse primer was designed to add the influenza virus hemagglutinin epitope tag (HA) and the *Not*I restriction site at the 3' extreme. Finally, the partial 5' end cDNA *RAI1 *clone (KIAA1820, Kazusa DNA Research Institute, Japan) was ligated with the ~1.5 kb fragment that contained the 3' end of the cDNA with *Bam*HI and *Not*I enzymes. The complete *RAI1 *cDNA clone was totally sequenced.

Three different point mutants were generated by site-directed mutagenesis by PCR with the kit QuickChange Site-Directed Mutagenesis XL Kit (Stratagene) utilizing the following primers: for the mutation A4685G, *Forward*: GCGACGACGACGGCAGCAGGTGCTG and *Reverse*: CAGCACCTGCTGCCGTCGTCGTCGC; for the mutation G5423A, *Forward*: CAAACATGAGTGCAACAAGGAGGCTC and *Reverse*: GAGCCTCCTTGTTGCACTCATGTTTG, for the deletion 3103delC, *Forward*: CTGCACAGGGCCCCCCAGGGACAGATGGAA and *Reverse*: CTTCCATCTGTCCCTGGGGGGCCCTGTGCAG. The deletion 2687delC was a spontaneous deletion found during the sequencing. The cDNA for the truncated protein R960X was generated by PCR with the following primers, *Forward*: CTGACCGGTGGCTGGAGGAC and *Reverse*: TGGTGGAATCCCCTGGAGCTCCTACTCC. The product contains a stop codon instead of base 2878. This fragment was subcloned into full-length *RAI1 *cDNA with the enzyme *Sac*I. All clones were verified by DNA sequencing.

Two C-terminal fragments of the RAI1 protein were generated using the following primers: for the fragment 1038-1915, *Forward*: GGGACAGATGGAAGGGGCTGG and for the fragment 1229-1915, *Forward*: GTCGGCCTTCATGGCGCCG. Both amplification reactions were performed with the same primer reverse used for generating the full-length clone, detailed above, which contains the HA tag and the *Not*I restriction site at the 3' extreme. PCR products obtained were fully verified by DNA sequencing.

For expression analysis, the cDNAs of *RAI1 *wild type and mutant forms were subcloned in pALTER-MAX vector (Promega).

#### Accession numbers

For KIAA1820 [GenBank: AB058723], Mouse Rai1 [GenBank: NM_009021 and Swiss-Prot: Q61818]. For Human RAI1 [GenBank: NM_030665 and Swiss-Prot: Q7Z5J4].

### Cell Culture

Neuro-2a and HeLa cells were grown in Dulbecco's Modified Eagle Medium supplemented with 10% fetal bovine serum, penicillin (100 units/ml), streptomycin (100 μg/ml) (Gibco by Invitrogen) at 37°C with 5% CO_2 _until 95% confluence was attained.

### Immunofluorescence and Western blot analysis

To study the expression of the proteins generated, Neuro-2a cells were transfected using Lipofectamine 2000 (Invitrogen), with the mouse plasmid pCR3.1 *Rai1-HA *and human constructs pALTER-MAX *RAI1-HA *wild type, *RAI1-HA *Q1562R, *RAI1-HA *S1808N, *RAI1 *R960X, *RAI1-HA *2687delC, *RAI1HA *3103delC, *RAI1HA *1038-1915 and *RAI1HA *1229-1915. All transfections were performed according to manufacturer's protocol.

For immunofluorescence, cells were fixed 36 h after transfection with 4% paraformaldehyde followed by permeabilization with 0.1% Triton X-100 in PBS. Subcellular localization of RAI1-HA wild type and mutant forms of the protein were detected using rabbit anti human RAI1 polyclonal antibody (1:200, ab58658 Abcam) whose epitope locates at the amino-terminal of RAI1. A secondary antibody conjugated to Alexa fluor 488 (1:500) was used. Cells were mounted in a medium with DAPI (Vector Laboratories).

For western blot analysis, total protein extracts were prepared 48 h after transfection. Cells were lysed in 100 μl of protein extraction and loading buffer (2% SDS, 2 M Urea, 10% Glycerol, 10 mM Tris pH 6.8, 0.002% Bromophenol Blue and 10 mM DTT) plus 1:200 protease inhibitor cocktail (SIGMA). The samples were homogenized passing 20 times through a syringe and warmed up to 95°C for 5 minutes. 25 μl of each cell lysate was loaded onto 10% SDS-polyacrylamide gels with Tris/glycine running buffer and transferred to a 0.2 μm polyvinylidene fluoride (PVDF, Bio-Rad) membrane. Immunodetection was performed using rabbit anti human RAI1 polyclonal antibody (1:1000, Abcam) and rabbit anti β-tubulin (1:1000, sc-9104 Santa Cruz). Results were visualized by chemiluminiscence.

The detection of murine Rai1-HA and proteins 1038-1915 and 1229-1915 was done with anti HA high affinity antibody (1:5000 for Western blot and 1:1000 for immunofluorescence clone 3F10, Roche).

### Reporter gene assays

Transient transfections in Neuro-2a cells were performed in 35 mm plates. The amounts of plasmid DNA used were according to manufacturer's protocol. GAL4-BD fusions of mouse and human *RAI1 *wild type and all of its mutant forms were co-transfected with the luciferase reporter plasmid pFR-Luc (Stratagene) and the β-Galactosidase expression plasmid pSV-β-Galactosidase (for normalization of the transfection efficiency). Forty eight hours post-transfection, cells were lysed and luciferase assays were performed with Luciferase Assay Kit (Stratagene), according to manufacturer's instructions. The Relative Lights Units (RLUs) were measured in duplicate in a Syrius Luminometer (Berthold Detection Systems).

β-Galactosidase activities were measured with 810 μl of β-Galactosidase buffer (60 mM Na_2_HPO_4_, 40 mM NaH_2_PO_4_, 10 mM KCl, 1 mM MgCl_2 _and 50 mM β-mercaptoethanol) for 50 μl of cell lysates plus 160 μl ONPG reagent (60 mM Na_2_HPO_4_,40 mM NaH_2_PO_4 _and 4 mg/ml ONPG (o-Nitrophenyl-β-D-Galactopyranoside). Incubations were performed at 30°C, reactions were stopped by adding 250 μl of Na_2_CO_3 _1 M. The absorbance was read at 420 nm. Each cell assay was carried out in duplicate.

## Authors' contributions

PCM, CAE, CPC, LC, JM and PK carried out the experiments. PCM, CAE, JIY and KW participated in the design of the study. PCM and KW conceived the work and drafted the manuscript. KW supervised and coordinated the study. All authors read and approved the final manuscript.
